# Attributable mortality to radon exposure in Galicia, Spain. Is it necessary to act in the face of this health problem?

**DOI:** 10.1186/1471-2458-10-256

**Published:** 2010-05-18

**Authors:** Mónica Pérez-Ríos, Juan M Barros-Dios, Agustín Montes-Martínez, Alberto Ruano-Ravina

**Affiliations:** 1 Department of Preventive Medicine and Public Health. University of Santiago de Compostela. Santiago de Compostela. Spain; 2 Epidemiology Section. Department of Public Health. Galician Regional Authority. Spain; 3 CIBER de Epidemiología y Salud Pública. CIBERESP, Spain; 4 Preventive Medicine Unit. Santiago de Compostela Clinic University Hospital. Santiago de Compostela. Spain

## Abstract

**Background:**

Radon is the second risk factor for lung cancer after tobacco consumption and therefore it is necessary to know the burden of disease due to its exposure. The objective of this study is to estimate radon-attributable lung cancer mortality in Galicia, a high emission area located at the Northwest Spain.

**Methods:**

A prevalence-based attribution method was applied. Prevalence of tobacco use and radon exposure were obtained from a previously published study of the same area. Attributable mortality was calculated for each of six possible risk categories, based on radon exposure and smoking status. Two scenarios were used, with 37 Bq/m^3 ^and 148 Bq/m^3 ^as the respective radon exposure thresholds. As the observed mortality we used lung cancer mortality for 2001 from the Galician mortality registry.

**Results:**

Mortality exclusively attributable to radon exposure ranged from 3% to 5% for both exposure thresholds, respectively. Attributable mortality to combined exposure to radon and smoking stood at around 22% for exposures above 148 Bq/m^3^. Applying the United States Environmental Protection Agency (EPA) action level, radon has a role in 25% of all lung cancers.

**Conclusions:**

Although the estimates have been derived from a study with a relatively limited sample size, these results highlight the importance of radon exposure as a cause of lung cancer and its effect in terms of disease burden. Radon mitigation activities in the study area must therefore be enforced.

## Background

Lung cancer is a serious Public Health problem. Tobacco is the main risk factor and radon exposure is the second one [1]. Radon is a colorless, odorless and tasteless gas with a half-life of 3.8 days. On decaying, its short-lived decay progeny emits radioactive alpha particles that cause lung cancer [2,3]. The relationship between this gas and lung cancer was first established in miners [4,5]. Epidemiologic studies observed later that persons exposed to elevated radon levels in their homes had a higher risk of developing lung cancer [3]. Radon gas has been declared a human carcinogen by the International Agency for Research on Cancer (IARC) [6]. These findings have led the United States Environmental Protection Agency (EPA)[7] and the European Commission[8] to set action levels at 148 Bq/m^3 ^and 200 Bq/m^3^, respectively, advising implementation of radon reduction measures at any home exceeding these thresholds. Accumulated evidence on the hazard posed by radon exposure has spurred many European countries, the USA and Canada to elaborate maps showing which geographic regions have elevated radon levels. WHO is also leading the International Radon Project, which has among its objectives to estimate the global burden of disease caused by radon exposure [9].

Galicia, the study area, has the highest indoor radon concentrations throughout Spain, due to the granitic nature of the subsoil and also because many homes in the countryside are built with granite. Different studies have shown that approximately 20% of all dwellings exceed the EPA action level [10,11].

In the context of decision making it is essential to know, albeit approximately, the impact that a given risk factor has on the mortality of a population [12]. Since atributable mortality is a good public health indicator that allows knowing the burden of disease caused by radon in lung cancer, the objective of this investigation is to calculate radon attributable mortality for Galicia, the highest risk area in Spain.

## Methods

### Setting

Galicia is a region in the Northwest of Spain, with a population of approximately 2,700,000 and an area of 28,000 sq. km. It is bordered to the North and West by the Atlantic Ocean, to the East by the remainder of mainland Spain, and to the South by Portugal. Former studies have indicated that it is an area with high radon emission levels [10,11,13].

### Method used and data sources

We used a method previously employed for the estimation of attributable deaths related to tobacco use [14] and environmental tobacco smoke [15].

Attributable lung cancer deaths (AM) are calculated as follows:

AM = OM × PAF, where OM (Observed Mortality) is the number of lung cancer deaths (of those 35 years and older) that occurred in Galicia in 2001 and PAF is the population-attributable fraction. PAF is a function of the exposure prevalence among cases (p) and Odds Ratio [16-18], and is denoted as . For the present study p is the proportion of subjects among lung cancer cases exposed to each of the risk factors analyzed, radon and tobacco. Since we consider two scenarios of radon exposure, 37 and 148 Bq/m^3^, and three categories for tobacco smoking (never smokers, ex-smokers and current smokers), six prevalences and six ORs are applied for each scenario. An ex-smoker was defined as any individual who had smoked a minimum of one cigarette per day but stopped at least one year before the interview for the study and a current smoker as an individual who has being smoking at least one cigarette per day in the last year. Those who quitted less than one year before the information was collected were classified as current smokers.

Prevalences and Odds Ratios were obtained from a population-based case-control study (with controls randomly selected) conducted in Galicia over the period 1992-1994 [10]. The objective of this study was to assess the relationship between radon exposure and lung cancer and included 163 cases and 241 controls (404 houses measured).

The observed mortality due to lung cancer in Galicia was obtained from the Galician Mortality Registry.

The 3 categories of smoking status were never-smokers, ex-smokers and current smokers. Risks were obtained using the SPSS 12.0 statistical package by means of unconditional logistic regression, where the dependent variable was the case or control status and the independent variables were the different categories of exposure to radon and smoking. As variables of adjustment, we considered age, sex, and family history of cancer.

## Results

The characteristics of the study subjects used to calculate radon attributable mortality are shown in Table 1. The geometric mean of radon exposure (geometric means were calculated because radon follows a log-normal distribution) was 66.4 Bq/m^3 ^for controls; and 75.4 Bq/m^3 ^for cases. The percentage of homes in the study area with concentrations above 148 Bq/m^3 ^(action level in the USA) is 19.4% which ranks Galicia as a risk region in terms of residential exposure. There were many more smokers among cases than among controls, and the number of packets smoked over a lifetime was also greater in these.

**Table 1:** Description of the study population used to obtain the risks and prevalences for the attribution model.

	Cases^a^	Controls^a^
SEX		
Males (%)	151 (92.6)	219 (92.9)
		
AGE (years)		
Median (SD)	65.7 (10.5)	57.7 (12.5)
		
EXPOSURE TO RADON^b^		
Geometric mean (GSD)	75.4 (2.4)	66.4 (3.0)
Median (pct 25, 75)	66.6 (40.7, 144.5)	51.8 (29.6, 118.4)
Number of homes (individuals) with concentrations above 37 Bq/m^3 ^(%)	125 (78.6)	155 (65.4)
Number of homes (individuals) with concentrations above 148 Bq/m^3 ^(%)	42 (26.4)	46 (19.4)
		
TOBACCO CONSUMPTION		
Never smokers (%)	14 (8.6)	98 (40.7)
Ex-smokers (%)	75 (46.0)	57 (23.7)
Current smokers (%)	74 (45.4)	86 (35.7)
Mean^c ^(SD)	24.2 (16.9)	7.1 (9.9)
Median^c ^(pct 25,75)	21.9 (14.6, 35.3)	7.1 (0.0, 10.9)
		
PRESENCE OF RADON MONITORING DEVICE		
Days (mean, SD)	155.3 (31.1)	150.9 (55.3)

^a^The total number of cases and controls was 404 individuals

^b^All radon measurements were adjusted by seasonality

^c^Calculated for current smokers. Thousands of packets smoked over a lifetime.

Exposure prevalences to tobacco and radon are shown in Table 2. The prevalence of radon exposure is higher above the 37 Bq/m^3 ^threshold and the opposite happens for the 148 Bq/m^3 ^one. Something similar occurs with smoking exposure for both thresholds. Odds Ratios are slightly higher when tobacco and radon exposure are combined compared with ORs calculated only for smokers.

**Table 2:** Prevalences and odds ratios^a ^used for estimating attributable mortality.^b^

	37 Bq/m3			148 Bq/m3		
	n	Prevalence	OR and 95% CI	n	Prevalence	OR and 95% CI
Non-exposed non-smokers	2	1.3%	1.00	8	5%	1.00
Non-exposed ex-smokers	23	14.5%	34.14 (5.81-200.42)	63	39.6%	48.65 (13.01-181.91)
Non-exposed smokers	9	5.7%	14.38 (2.28-90.55)	46	28.9%	29.95 (8.12-110.48)
Exposed non-smokers	12	7.4%	2.50 (0.47-13.46)	6	3.9%	7.39 (1.68-32.56)
Exposed ex-smokers	51	32.1%	44.88 (8.13-247.63)	11	6.9%	24.07 (5.24-110.52)
Exposed smokers	62	39.0%	43.23 (7.96-234.92)	25	15.7%	53.41 (12.88-221.49)

^a ^Adjusted for sex, age, and family history of cancer.

^b ^Source: Barros-Dios JM, Barreiro Carracedo MA, Ruano-Ravina A, Figueiras A. Exposure to residential radon and lung cancer risk in Spain: a population-based case-control study. Am J Epidemiol. 2002. 156 (6): 548-55.

Table 3 shows the estimated attributable mortality in percentage terms and absolute numbers. In both scenarios, mortality attributed to causes other than smoking and radon ranged from 6.5% to 8%. Mortality exclusively attributable to smoking was 67% for those exposed to over 148 Bq/m^3 ^whereas mortality exclusively attributable to radon was 4.5% and 3.3% for subjects exposed to over 37 Bq/m^3 ^and 148 Bq/m^3^, respectively. In the 148 Bq/m^3 ^scenario it can be observed that radon exposure participates in 25% of all lung cancer deaths and that the 22% of all deaths can be attributed to the simultaneous exposure to radon and smoking. These figures are much higher for the 37 Bq/m^3 ^scenario since most of the population is exposed to higher radon concentrations.

**Table 3:** Percentage of lung cancer mortality in Galicia attributable to exposure to radon and smoking

	37 Bq/m^3^		148 Bq/m^3^	
	
	Attributable deaths	Attributable number	Attributable deaths	Attributable number
Non-exposed never-smokers	6.66%	87	7.98%	105
Non-exposed ex-smokers	14.07%	185	38.78%	509
Non-exposed current smokers	5.30%	70	27.93%	367
Exposed never-smokers	4.50%	59	3.29%	43
Exposed ex-smokers	31.38%	412	6.61%	87
Exposed current smokers	38.09%	500	15.41%	202
Total	100%	1313	100%	1313

## Discussion

This is the first study in Spain and one of the few undertaken in Europe that estimates lung cancer mortality attributable to radon. The main result is that, while mortality exclusively attributable to radon is low, i.e., in the order of 3%-5%, mortality attributable to the combined exposure to radon and smoking is far higher, 22% for ever smokers exposed to over 148 Bq/m^3^. The attributable mortality to individuals exposed to over 37 Bq/m^3 ^is higher, and results point out that a high number of lung cancer deaths could be prevented with radon mitigation measures along with smoking cessation.

These results are in line with those reported by other studies. A study conducted in West Germany [19] observed that, with a geometric mean radon concentration of 40 Bq/m^3^, the attributable fraction ranged from 7% to 22% for male non-smokers and from 5% to 7% for smokers, suggesting that radon might be more important in relative terms in non smokers compared with smokers. The authors also estimated that 7% of all lung cancer deaths could be attributed to radon. In 1987, the German Radiation Protection Commission estimated that 4%-12% of lung cancer mortality could be due to inhalation of residential radon [20]. Two studies have been recently published estimating radon attributable mortality for lung cancer in England and France. In England around 3.3% of lung cancer cases are attributable to radon (with a mean radon concentration of 21 Bq/m^3^)[21] and the French study showed that radon causes from 2 to 12% of all lung cancer deaths [22].

In the opinion of Lubin and Boice [23], 14% of lung cancer deaths are attributable to radon (with "exposed" defined as being from 2 Bq/m^3 ^upwards). Eliminating exposures above 296 Bq/m^3 ^and 148 Bq/m^3 ^would prevent only 2% and 5% of lung cancer cases, respectively. Approximately 80% of deaths attributed to radon occur among smokers. This attributable mortality is very similar to that calculated by Puskin and Yang [24]. Another study, conducted by Leenhouts and cols [25] indicates that higher exposure to radon among the Swedish versus the Dutch population is reflected in the percentage of lung cancer attributable to radon, i.e., 25% versus less than 10%. Among males, the percentage exclusively attributable to radon is 2% in Holland and 17% in Sweden (for women these percentages are 6% and 24%, respectively). In both countries, radon and/or tobacco account for 80% of all lung cancer deaths. These differences highlight the fact that calculating attributable radon mortality to a whole country with large differences in residential radon exposure would be rather misleading since radon is usually a public health problem in specific areas, which is what happens in Spain.

It is normal for attributable mortality above 37 Bq/m^3 ^to be higher than that above 148 Bq/m^3^, since the number of exposed subjects is higher. The figure of 37 Bq/m^3 ^was chosen because earlier studies in the same area had already indicated a significative risk of lung cancer as from this radon level, along with evidence of an interaction with smoking [10]. The 148 Bq/m^3 ^threshold was chosen because it is the designated EPA action level. It is also very important to keep in mind that although having defined, for practical reasons in one scenario, those individuals under 148 Bq/m^3 ^as non exposed, they are still subject to a considerable risk of lung cancer due to radon exposure. Choosing two exposure levels allows for comparison of attributable mortalities and also highlights the fact that there is no safe radon-exposure level, as shown by the 4.5% of deaths among non-smokers exposed to over 37 Bq/m^3^. When radon concentration is higher than 148 Bq/m^3^, up to 25% of lung cancer deaths (among smokers, ex-smokers, and non-smokers alike) could probably be prevented with radon reduction interventions, which translates as 332 lung cancer deaths in the study area, almost one per day in 2001. Nevertheless, Denman and cols [26] have indicated that, even though radon affected areas have been defined by the National Radiological Protection Board as those with over 1% of homes above the action level, radon mitigation activities could only be cost-effective in areas with 5% of homes above 200 Bq/m^3^. At all events, Galicia more than fulfills this requirement, with 17.7% of homes exceeding 200 Bq/m^3^.

The interaction that exists between radon and tobacco use (seen as additive by some and multiplicative by others) [27-29] has been used by health authorities as a means to persuade people to quit smoking, and the data yielded by this study seem to support this practice. Nevertheless, the fact that most of the Galician population have no idea about radon and its effects, does not contribute to address the problem posed by radon exposure. Counteracting this ignorance is the recent WHO initiative [9], aimed at raising the awareness of governments, authorities and, ultimately, citizens, to radon and the related health risk, so that authorities take (and citizens demand) the necessary measures. One of the actions proposed is the inclusion of radon mitigation guidelines in building technical regulations, something that is simple to implement where new buildings are being constructed but more difficult in the case of existing buildings. Many countries have already introduced mandatory guidelines to prevent or reduce radon entry in buildings. Galicia has passed a law in January 2009 compelling all new dwellings to be protected against radon entry [30].

This study has a number of strengths and one of them is that it is the first to estimate radon-attributable lung cancer mortality in Spain. Another advantage is that the data obtained for constructing the attribution model came from a study performed in the same area to which such mortality is sought to be attributed and where controls were randomly chosen from the census and the risks adjusted for sex, age, and family history of cancer. Although uncertainty is always present in epidemiologic research and is even higher in attribution studies, we do not believe that this might be a problem with our calculations because the parameters used in the attribution formula (prevalences and ORs) have been obtained using reliable methods and are representative of the study area.

Our study also suffers from limitations. Two are related to the case-control study used to estimate prevalence of exposure. The number of cases was low and also the sample size; nevertheless, other cross-sectional studies undertaken in Galicia support the estimated smoking prevalence [31]. Another limitation is that it assumed uniform exposure to a given radon concentration throughout lifetime, something that is not true because people are likely to move home. Other studies take this mobility into account but while mobility is an important factor in other countries [32], in Galicia individuals tend to remain in the same home for many years. Indeed, in the study from which the data came, two thirds of all subjects had lived for over 20 years in the dwellings where radon was measured, with no significant differences between cases and controls [10]. The fact that the method assumes lung cancer incidence to be equivalent to mortality could be considered as a problem, but this assumption is easy to make for this particular cancer owing to its high lethality and the impossibility of taking into account the length of time during which ex-smokers have not smoked. A further possible disadvantage might be that the method used is different to that employed by other studies or the BEIR VI model [2] and thus hinders comparability of results, but in our opinion the used method is the best suited to the available data, due to its applicability to the studied region. One model could have been used to estimate radon attributable deaths using two cut-points on 37 Bq/m^3 ^and on 148 Bq/m^3^. This was not done since for each of the three exposure categories created three Odds Ratios would be needed (never smokers, ex-smokers and current smokers) having finally nine ORs and nine prevalences. The small sample size of our study would therefore render inaccurate estimations when using many categories. The only difference between the two models is the radon cut-points, being the other variables included exactly the same.

## Conclusions

Two conclusions can be drawn from the results of this study, although these are limited due to the low sample size. First, mortality exclusively attributable to the effect of radon is -as expected- quite low, between 3%-5%. Second, mortality attributable to combined exposure to radon and smoking is considerably high in Galicia, around 22% (with radon participating in 25% of all lung cancer deaths), confirming once again the problem posed by this gas in this north-western region of Spain. This is an important health problem affecting the Galician population and it calls for a deep involvement of national and regional authorities.

## Competing interests

The authors declare that they have no competing interests.

## Authors' contributions

ARR and JMBD had the idea of the paper and revised the results. MPR selected and performed the method to attribute mortality and did all the calculations. MPR and ARR wrote the manuscript and performed the literature review. All authors revised critically the manuscript and made intellectual contributions to it.

## Pre-publication history

The pre-publication history for this paper can be accessed here:

http://www.biomedcentral.com/1471-2458/10/256/prepub
